# The impacts of Extra-tropical ENSO Precursors on Tropical Pacific Decadal-scale Variability

**DOI:** 10.1038/s41598-020-59253-3

**Published:** 2020-02-20

**Authors:** Yingying Zhao, Emanuele Di Lorenzo

**Affiliations:** Program in Ocean Science and Engineering, Georgia Institute of Technology, Atlanta, USA

**Keywords:** Physical oceanography, Physical oceanography

## Abstract

Off-equatorial wind anomalies on seasonal timescales from both the North and South Pacific, known as “precursors” of the El Niño Southern Oscillation (ENSO), have been shown to independently trigger the ENSO feedbacks in the tropics and its teleconnections to the extra-tropics. However, the impacts of ENSO precursors on Tropical Pacific Decadal-scale Variability (TPDV) is still not well understood and quantified. We show that the dynamic sequence from extra-tropical ENSO precursors to ENSO (tropics) to extra-tropical ENSO teleconnections is not only important for ENSO, but acts as a primary mechanism to filter (e.g. reddening) the low-frequency variability of the seasonal precursors into the decadal-scale variance of the Pacific basin, accounting for the largest fraction of the TPDV (~65%) and its phase. This process, which contrasts previous theories advocating for a TPDV generated internally in the tropics (e.g. ENSO residuals), is inherently unpredictable and not well reproduced in climate models and raises challenges for understanding and predicting the role of internal TPDV in future climate change scenarios.

## Introduction

Low-frequency variability of tropical Pacific climate on decadal and longer timescales, referred to in the literature and here as “decadal variability”, is known to influence large-amplitude changes in marine ecosystems, climate and weather extremes over the Pacific Ocean, Asia and the Americas with important societal impacts^1–4^. Furthermore, tropical Pacific decadal variability (TPDV) has been implicated as a driver of the global warming hiatus^5–8^, yet the mechanisms controlling its phase and predictability remain unclear.

**Figure 1:**
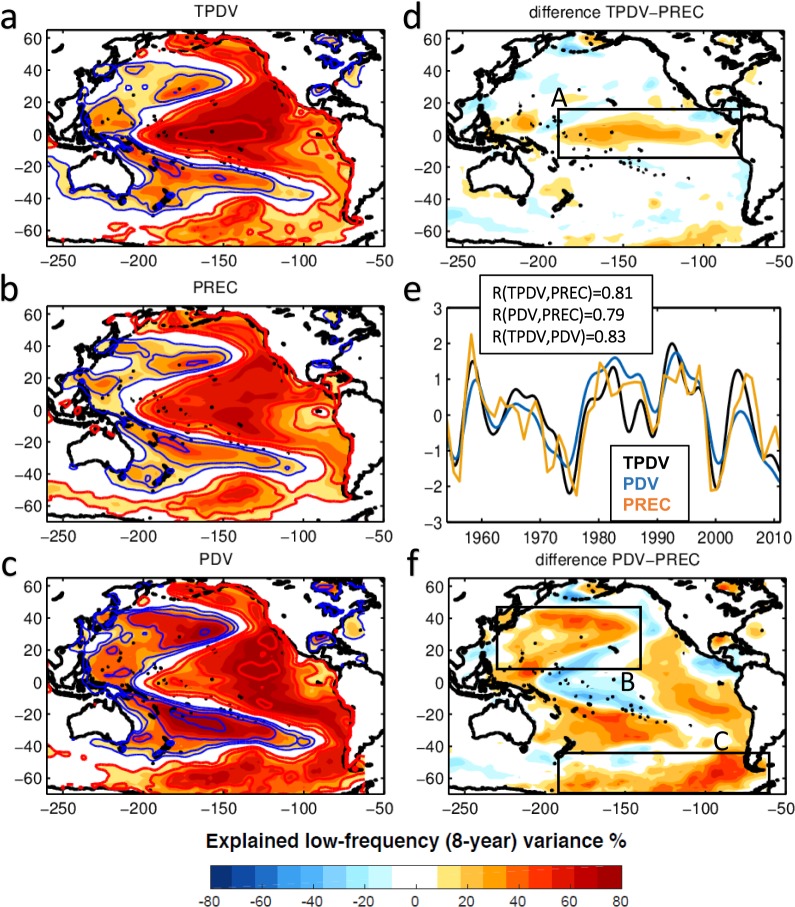
Pacific decadal variance explained by tropical and extra-tropical variabilities. Correlation square (R^2^) between 8-year lowpass SSTa and (**a**) tropical Pacific decadal variability index (TPDV), (**b**) decadal variability index of South and North extra-tropical precursors (PREC), and (**c**) Pacific decadal variability index (PDV). The contours in the maps show the correlation coefficients (R). The red and blue lines show the positive and negative values, respectively. Correlation of R = 0.62 (R^2^ = 38% of explained variance) or higher are significant at the 95%. (**d**) Difference map of variance explained by TPDV minus that explained by PREC. (**f**) Difference map of variance explained by PDV minus that explained by PREC. Black boxes in (d) and (f) highlight the main differences of the explained variance. (**e**) Comparison of the TPDV (black line), PREC (yellow line), and PDV (blue line) indices. Also indicated are the correlation coefficients between the indices, which pass the 95% confidence level.

The spatial footprint of TPDV in global sea surface temperature anomaly (SSTa) can be extracted by correlating the time series of the dominant mode of 8-year lowpass SSTa in the tropical Pacific [10°S–10°N] with global SSTa (Fig. 1a) (*see Methods*). This approach is similar to previous characterization of basin-scale Pacific decadal variability (PDV), which uses the dominant mode of lowpass SSTa over the entire basin^9^ (Fig. 1c). The TPDV and PDV patterns are not independent and exhibit very similar structures in the squared correlation explaining ~65% of the Pacific decadal variance (Fig. 1a vs. 1c). A comparison of the time series associated with the modes, hereinafter the TPDV and PDV indices (*see Methods for exact definition*), show significant correlation (R = 0.83, > 99% confidence level) suggesting that the tropics plays a key role in shaping the basin-scale Pacific decadal variance (Fig. 1e). This is further confirmed by the fact that the dominant modes of Pacific low-frequency variability extracted independently over the North and South Pacific, such as the Pacific Decadal Oscillation^10^, the North Pacific Gyre Oscillation^11^, and the South Pacific Decadal Oscillation^12^, are not independent in the low-frequency limit and all exhibit the spatial and temporal structure of the TPDV pattern^13^. Given that the tropics is the main bridge between the North and South Pacific climate variabilities, it is clear that tropical dynamics synchronize the low-frequency decadal-scale variance in the two hemispheres.

While sources of TPDV have been identified both within and outside the tropics^14^, a clear separation of the dominant processes that energize TPDV and its phase is hindered by the wide-range of model-dependent ENSO and TPDV mechanisms. In the tropics, ENSO and its teleconnections are known to transfer tropical interannual variance into the extra-tropical atmosphere^1,15^, which in turn energizes the decadal ocean modes of the North and South Pacific^16–21^. For this reason, the PDV pattern has been often described in terms of an ENSO-like decadal variability^9^. Simulations with coupled ocean-atmosphere models of ranging complexity all show that the basic structure of the PDV and TPDV patterns can be reproduced simply by accounting for variability in the tropical Pacific associated with ENSO^22,23^. However, ENSO itself can be energized by precursors dynamics that are initiated in both the tropics and extra-tropics. Several studies show that prior to ENSO, stochastic variability of the equatorial winds, known as westerly wind bursts^24–27^, and tropical Atlantic teleconnections to the Pacific^28,29^, can activate the ocean-atmosphere positive feedbacks that energize ENSO and its teleconnections. These dynamics allow to transfer the stochastic energy of the tropical precursors into basin-scale variance. However, ENSO precursors have also been identified in the extra-tropics in connection to seasonal modulations of the off-equatorial trade winds^30–35^. Recent studies that examine the growth, peak and decay of the TPDV pattern argue that ENSO dynamics in the tropics alone cannot explain the growth phase of the TPDV and that extra-tropical stochastic forcing is important in driving TPDV^13^.

Using observational reanalysis, this study aims at quantifying the combined impacts of extra-tropical ENSO precursors from both the South and North Pacific, and show that these precursors are not only important for tropical variability on ENSO timescales but also control a large-fraction of the low-frequency tropical variability.

## Mechanistic Hypothesis for generating Tropical Low-Frequency Variability

The extra-tropical ENSO precursor dynamics can be initiated independently in both the South and North Pacific through anomalies in off-equatorial trade winds. For example, a reduction in the North Pacific off-equatorial trades in the boreal spring can trigger the winds-evaporations-SST (WES) thermodynamic feedback^36^ resulting in ocean-atmosphere coupled anomalies that propagate into the tropics and can initiate ENSO in summer and fall. This process, first described in the context of the seasonal footprinting mechanism^33^, leads to coupled ocean-atmosphere Meridional Modes that have been shown to influence ENSO and tropical variability from both hemispheres independently^28,37–40^. The weakening of the off-equatorial winds can also influence or trigger the development of ENSO-like variability by two ways. One is through modulating the strength of the sub-tropical cells and meridional heat transport into the equatorial thermocline^25,32,35,41^. The other is by exciting off-equatorial Rossby waves^30,34^, which propagate thermocline anomalies to the equatorial western boundary. The reflection of these waves as eastward Kelvin waves along the equator is known to trigger the ENSO feedbacks by modulating upwelling (e.g. the thermocline feedback). Although observational evidence for each of these precursor pathways exists, a clear attribution of the TPDV variance that is generated from the extra-tropical ENSO precursor remains elusive because of the short observational record and the fact that coupled ocean-atmosphere models are inconsistent in the representations, and relative strength, of the ENSO precursors^42^.

**Figure 2:**
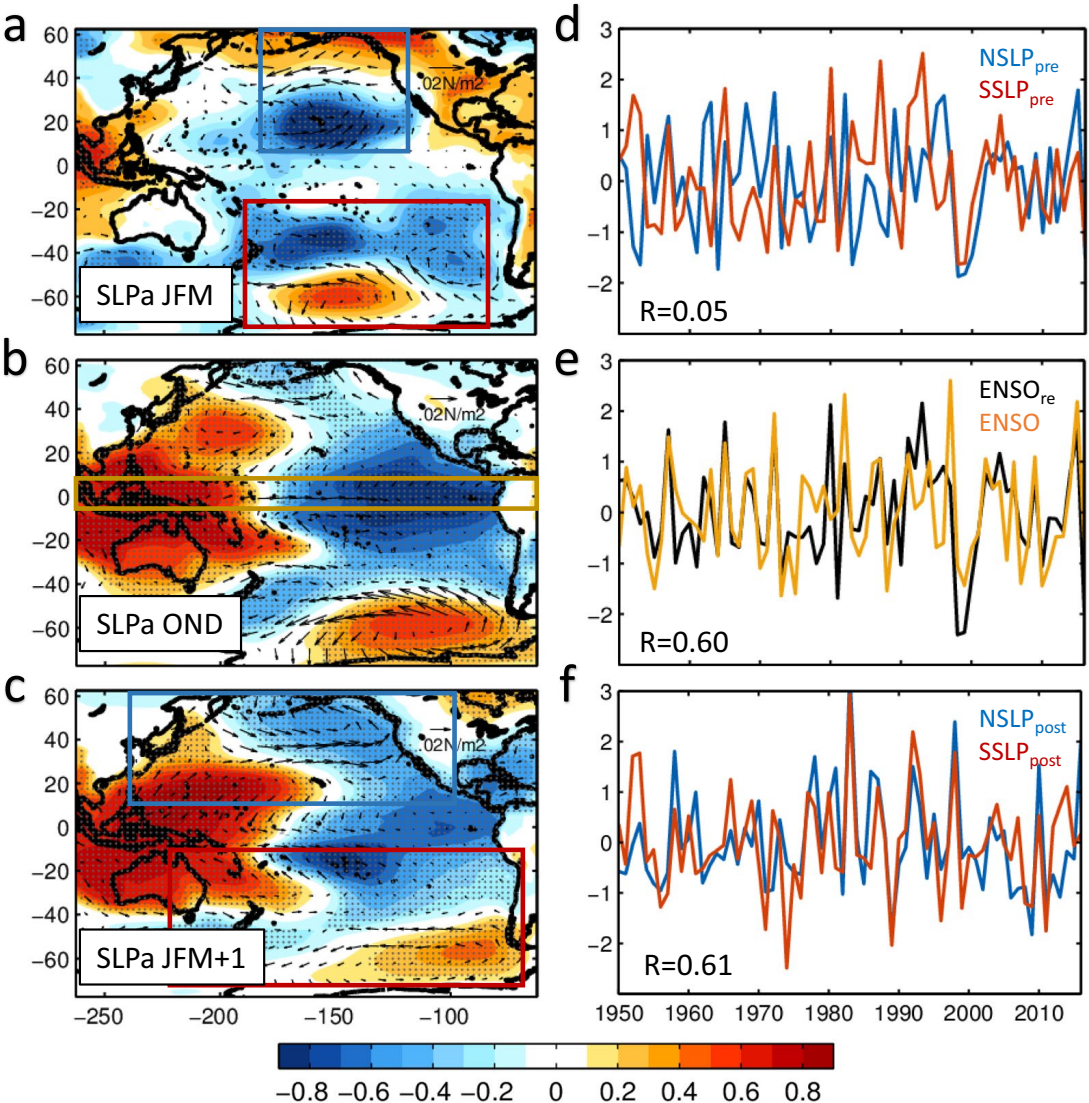
Atmospheric ENSO precursor, ENSO and ENSO successor patterns and the corresponding indices. Patterns are obtained by correlating NDJ ENSO index with (**a**) NCEP SLPa in JFM, (**b**) NCEP SLPa in OND, and (**c**) NCEP SLPa in the following JFM. Note that the correlations in figure (**a**) are multiplied by 2. Grey dots show the regions that pass the 90% confidence level. Wind vectors are obtained by regressing NDJ ENSO index onto (**a**) NCEP wind stress anomalies in JFM, (**b**) NCEP wind stress anomalies in OND, and (**c**) NCEP wind stress anomalies in the following JFM. The boxes in figure (**a**–**c**) show the domains for calculating the corresponding indices in figure (**d**–**f**), respectively. More details are described in Method. (**d**) Comparison of *NSLP*_*pre*_ index (blue line) and *SSLP*_*pre*_ index (red line). (**e**) ENSO index (yellow line) and ENSO_*re*_ index (black line): ${{\rm{ENSO}}}_{re}=0.5\times NSL{P}_{pre}+0.5\times SSL{P}_{pre}.$ (**f**) *NSLP*_*post*_ index (blue line) and *SSLP*_*post*_ index (red line). Also indicated are the correlation coefficients between the indices. Correlation coefficients in figure (**e**,**f**) pass the 99% confidence level.

Independently of the specifics of the extra-tropical precursor dynamics, the seasonal patterns of the wind precursors are isolated by correlating an index of ENSO in the boreal winter season November-December-January (NDJ) with the preceding winter season January-February-March (JFM) sea level pressure anomalies (SLPa) (Fig. 2a) (*see Methods for definition of ENSO index*). These wind precursors are characterized by a dipole pattern in sea level pressure anomalies (SLPa) in both the South and North Pacific, with low pressure close to the tropics corresponding to the weakening of the sub-tropical trade winds. Despite the spatial symmetry of the SLPa with respect to the equator, the variability of the North and South Pacific atmospheric precursors is primarily independent^33,38^. This is confirmed by the insignificant correlation (R = 0.05) (Fig. 2d) that exists between a seasonal index of the north and south SLPa precursors, here and after the *NSLP*_*pre*_ and *SSLP*_*pre*_ indices, computed by projecting the northern and southern precursor’s correlation patterns onto the SLPa in JFM (*see Methods*). The fact that these two extra-tropical precursors are uncorrelated further supports the idea that these wind anomalies are associated with seasonal stochastic variability that is independent of ENSO, given that any ENSO-related signal would be coherent within the entire subtropics. Furthermore, a cross-correlation analysis between each of the seasonal precursor indices and ENSO NDJ index shows significant correlations (R~0.4–0.5, >99% confidence level) only when the precursor indices are leading ENSO by 10 months (*see* Supplementary Fig. S7). The lack of cross-correlation when the ENSO index is leading provides more evidence that the precursor indices are largely independent of ENSO.

A linear model to reconstruct the ENSO NDJ index from the two precursors *ENSO*_*re*_ = 0.5×*NSLP*_*pre*_ + 0.5×*SSLP*_*pre*_ (*see Methods*) shows that the combined JFM stochastic atmospheric variability from both hemispheres captures a large and significant fraction of the NDJ ENSO variability (R = 0.6, >99% confidence level) (Fig. 2e) with a ~10months lead. Following the seasonal peak of ENSO, which is characterized in the SLPa by a weakening of the Walker Cell in the tropics (Fig. 2b) with low (high) SLPa in the eastern (western) tropical Pacific, the following JFM (Fig. 2c) exhibits the classical ENSO teleconnection pattern in both hemispheres with an intensification of the North Pacific Aleutian Low and of the South Pacific Oscillation patterns (Fig. 2c). The oceanic response to these ENSO teleconnections is known to energize the North and South Pacific decadal variability oceanic modes^16–21^. Given the role of ENSO in driving these teleconnection patterns, indices of the north and south teleconnection patterns in JFM + 1 (*see Methods*) are no longer independent and share a significant correlation (R = 0.61, > 99% confidence level). A similar analysis of the ENSO precursor and teleconnection dynamics can be conducted using the NOAA ERSSTa v3 reanalysis (*see Methods and* Supplementary Fig. S3). The resulting oceanic ENSO precursor patterns are consistent and significantly correlated (R~0.8–0.9) with the optimal perturbation patterns of ENSO obtained by using more rigorous approaches like Linear Inverse Models (LIMs) and multivariable linear regression (MLR) (Supplementary Figs. S1 and S2 and Supplemental Material). In addition, to verify the robustness of this framework, we reproduce similar analyses with the Hadley data and obtained the same results (*see* Supplementary Figs. S4 and S5). Consistent with previous findings^38,43^, the timeseries of the JFM SSTa precursors variability for the North and South Pacific, here and after referred to as the *NSST*_*pre*_ and *SSST*_*pre*_ indices, show insignificant correlation with R = 0.08 for the ERSST (Fig. S3) and R = 0.17 for Hadley (Fig. S5), suggesting that both the southern and northern precursors drive an independent fraction of the ENSO variance. However, after ENSO peaks, the teleconnection responses are coherent in both hemispheres.

**Figure 3:**
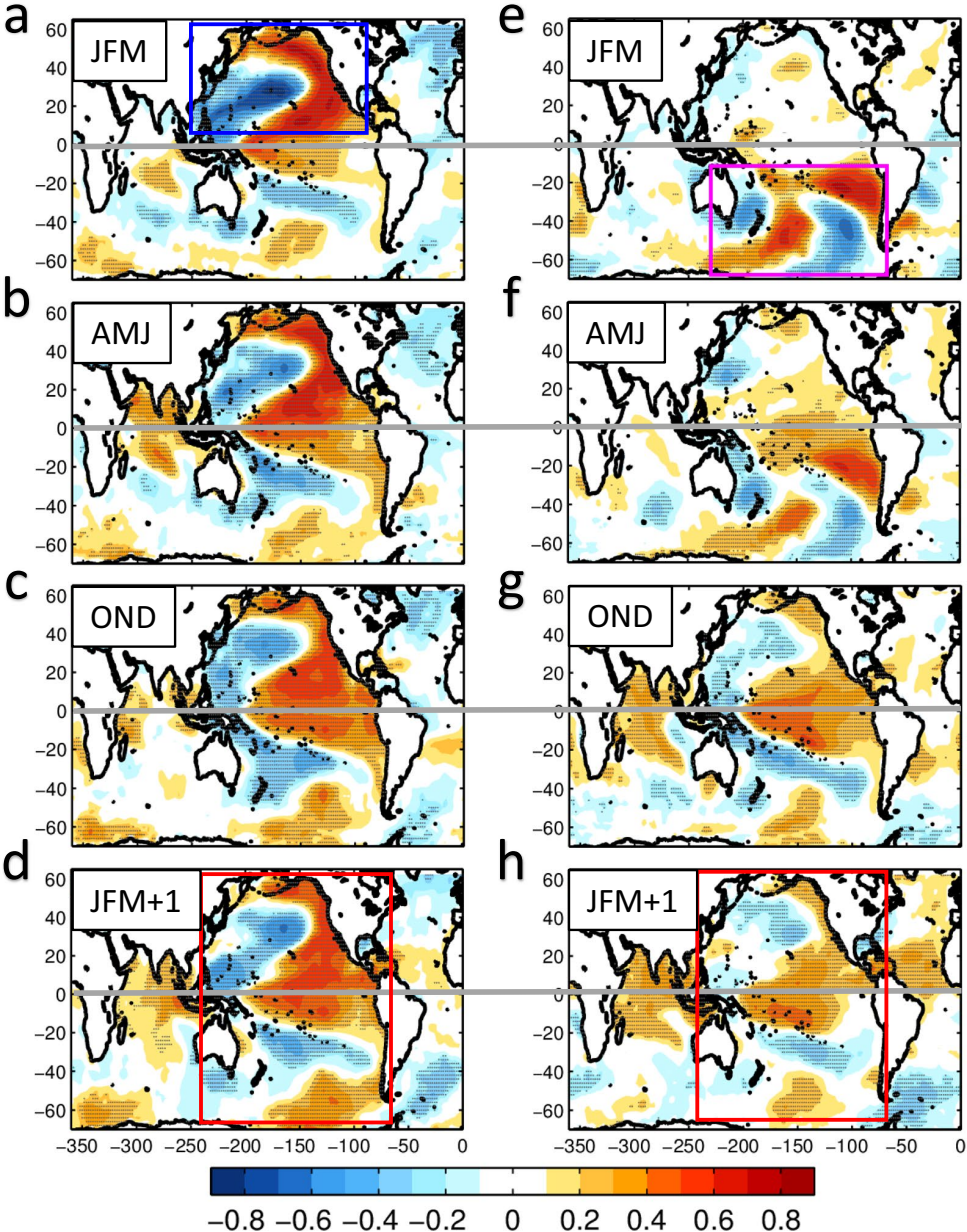
Seasonal progression of the ENSO North Pacific precursor (the left column) and ENSO South Pacific precursor (the right column). Correlation maps between *NSST*_*pre*_ index and seasonal SSTa in (**a**) JFM, (**b**) AMJ, (**c**) OND and (**d**) the following JFM; correlation maps between *SSST*_*pre*_ index and seasonal SSTa in (**e**) JFM, (**f**) AMJ, (**g**) OND and (**h**) the following JFM. Grey dots show the regions that pass the 90% confidence level. The boxes in figure (**a**,**e**) show the initial northern and southern precursor patterns, respectively. The boxes in figure (**d**,**h**) show the ENSO teleconnection patterns evolved from the northern and southern precursor patterns, respectively.

By energizing ENSO and its teleconnections, the extra-tropical precursor dynamics transfer the seasonal variance originating in one of the hemispheres (e.g. North or South Pacific) into the other hemisphere with the typical ENSO-like interhemispheric pattern. This can be clearly visualized by examining the seasonal progression of the ENSO precursors in the SSTa field (Fig. 3). The seasonal evolution of the precursors from the North Pacific is visualized by correlating the JFM *NSST*_*pre*_ (from ERSST) with the seasonal SSTa in JFM, AMJ, OND and the following JFM (first column in Fig. 3). Similarly, the evolution of the South Pacific precursor is identified using the same correlation analysis for the *SSST*_*pre*_ (second column in Fig. 3). We find that in JFM, the patterns of the SSTa precursors are distinct and independent between the North and South Pacific. However, as the ENSO precursors progress in the season (e.g. AMJ, OND), the development of ENSO (AMJ, OND) makes the patterns more similar and symmetric with respect to the tropics with the classical ENSO-like shape. This seasonal progression allows to transfer the stochastic SSTa variance from one hemisphere into coherent basin-scale variance that has an ENSO-like spatial footprint.

**Figure 4:**
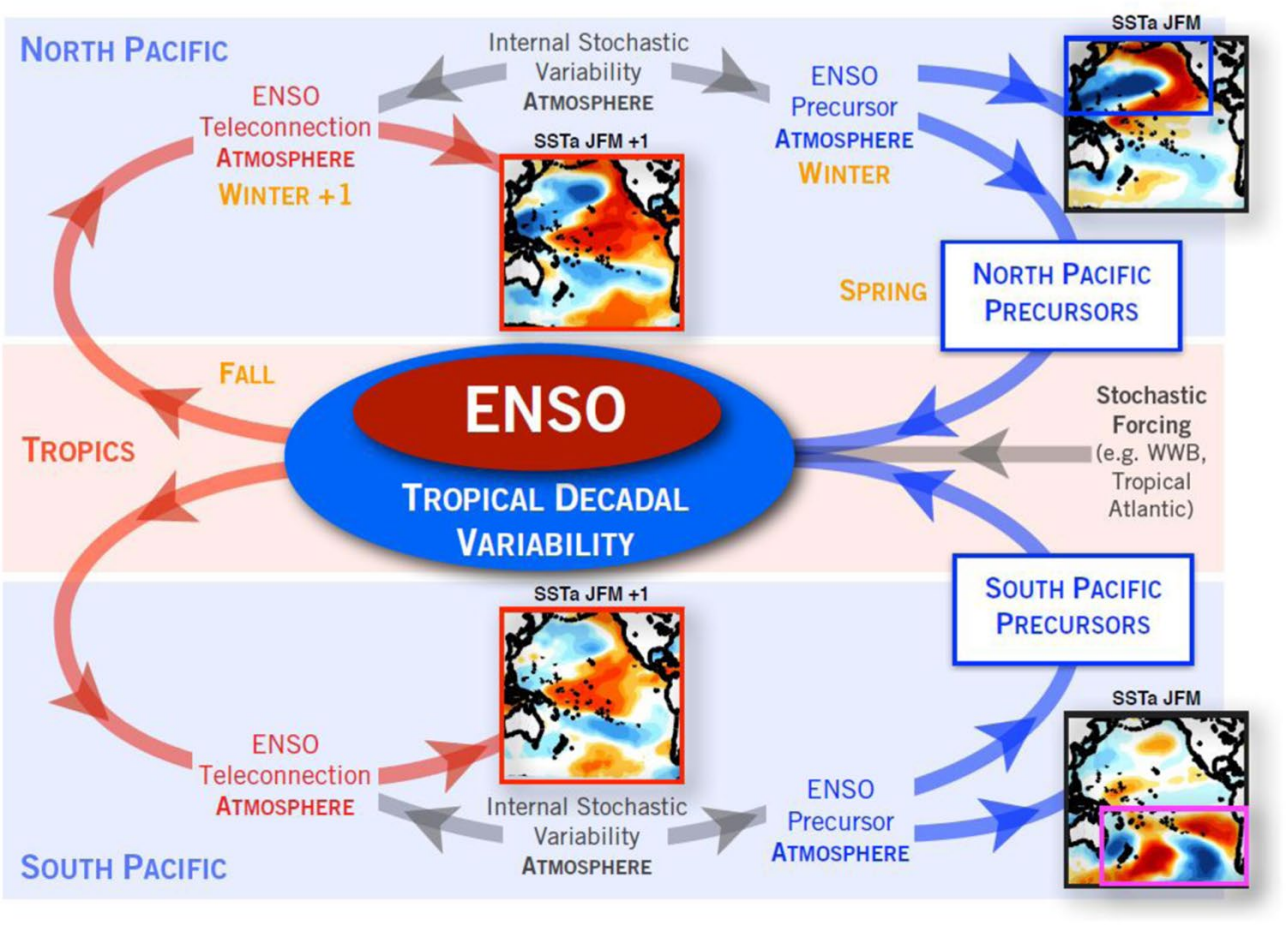
Framework of tropical Pacific Decadal Variability. The gray path shows how high-frequency stochastic variabilities in the atmosphere influence ENSO systems. The blue path shows the growing phase of the TPDV, which is the sequence ENSO precursors (extra-tropics) → ENSO (tropics). The red path shows the decaying phase of the TPDV: ENSO (tropics) → ENSO teleconnections (extra-tropics). The SSTa JFM and JFM + 1 maps in the North Pacific (South Pacific) region are the same as Fig. 3a,d (Fig. 3e,h), which represent the ocean expressions of the northern and southern ENSO precursors at JFM and their seasonal progression at JFM + 1.

We hypothesize that the interaction sequence from extra-tropics (ENSO precursors) to tropics (ENSO) to extra-tropics (ENSO teleconnections) acts as a mechanism to filter (e.g. reddening) the low-frequency variability of the seasonal precursors into TPDV. This sequence of teleconnections is summarized in the schematic of Fig. 4. In JFM, the seasonal stochastic variability in the extra-tropical atmosphere energizes the ENSO precursor dynamics in both the North and South Pacific independently (Fig. 4, blue path). This independence is also reflected in the uncorrelated structures of the north and south SSTa JFM precursor patterns (Fig. 4, SSTa JFM maps). Following the activation of the ENSO feedbacks and teleconnections (Fig. 4, red path), the successor patterns in the following winter (Fig. 4, SSTa JFM + 1), obtained by correlating the North and South Pacific SSTa JFM precursor indices with the SSTa in the following JFM, show spatial structures that are spatially correlated with the ENSO-like basin scale pattern. This sequence leads to an inherent memory of the Pacific climate ~ 1–2 year^13^. This memory allows to extract the low-frequency variability of the precursor forcing (e.g. by integrating the forcing, see next section) and to project that into the ENSO-like decadal variability pattern.

## The Impacts of ENSO Precursors on TPDV and PDV

To quantify the contribution of the seasonal extra-tropical precursors to TPDV and PDV, we test how much of the TPDV and PDV variance can be explained by the reddening of the precursor indices. Specifically, we develop an index of the low-frequency variability associated with the reddening of the seasonal precursors by integrating the *ENSO*_*Precursors*_ = 0.5×*NSST*_*pre*_ + 0.5×*SSST*_*pre*_ with an auto-regressive model of order 1 (AR-1) following the Hasselmann (1976) model^44^ (*see Methods*). Before running the AR-1 model we confirmed that the low-frequency modulation in the strength of the precursor indices are also independent (Fig. S8, R = 0.03 between the 8-year lowpass precursors indices). This provides further evidence that the decadal variance of the precursors is unlikely caused by tropical Pacific sources because it would generate coherent signal across the extra-tropics. We also checked that the *ENSO*_*Precursors*_ timeseries has insignificant lag-1 auto-correlation (Fig. S7) and is therefore consistent with the AR-1 model assumption that the forcing term is “white noise” (e.g. no auto-correlation). The timeseries obtained from the AR-1, defined as the PREC index (Fig. 1e), captures a substantial and significant fraction of the TPDV and PDV variability with correlations of R~0.8 (>99% confidence level), implying that the decadal phase of TPDV and PDV is linked to low-frequency modulations in the strength of extra-tropical precursors. In the AR-1 model, the memory timescale *τ* is a free parameter that needs to be set. Given that the teleconnection sequence between extra-tropics/tropics/extra-tropics (hypothesis Fig. 4) has an inherent timescale of ~ 1–2 year^13^, we explored using values in this range. We find that the correlations between PREC index and the TPDV/PDV indices is not very sensitive within this range of memory timescales (R = 0.78–0.82) and has a maximum *τ* = 1.2 *years*.

Using the PREC index, we can extract the fraction of spatial decadal variance explained by the precursors by a map of the correlation square between the PREC index and the 8-year lowpass SSTa (Fig. 1b). The PREC decadal pattern captures the main spatial features of the TPDV and PDV and explains on average 60% of the basin-scale variance. A difference map of the TPDV minus PREC variance (Fig. 1d) suggests that the largest fraction of basin-scale TPDV originates from the low-frequency variability of the ENSO precursors. A similar difference map between the PDV and PREC variance pattern reveals two large fraction of variance that are not explained by the PREC (Fig. 1f). The first one is located in the Kuroshio Oyashio Extension (KOE) region in the North Pacific and the second one in the Antarctic Circumpolar Current (ACC) in the South Pacific. This result is consistent with previous studies showing that both these regions have significant decadal variability that is independent of the tropical Pacific and generated in the higher latitudes.

While these results outline the important role that Pacific extra-tropical ENSO precursors play in TPDV and PDV, other studies have identified the tropical Atlantic as an important ENSO precursor. Similar to the analysis presented above, we developed an index of the seasonal tropical Atlantic SSTa precursor (*ASST*_*pre*_) in the JFM prior to ENSO (*see Methods*). The *ASST*_*pre*_ index has significant correlation with the NDJ ENSO index (R = 0.45, >99% confidence level) and explains the same amount of variance as the individual North and South Pacific precursors indices on interannual timescales (*see* Supplementary Fig. S6a,b). However, when we apply the AR-1 model to *ASST*_*pre*_ the correlations with the TPDV index become insignificant (R = 0.23) (*see* Supplementary Fig. S6c), in contrast to the PREC index (R~0.8, >99% confidence level). This indicates that while both Pacific and Atlantic precursors contribute to ENSO variability on interannual timescales, the decadal variability of the tropical Pacific is predominantly linked to the Pacific extra-tropical precursor and the dynamics of exchange between extra-tropics and the tropics.

Taken together, the dynamics of interaction of the North and South Pacific precursors with ENSO, suggest that a large fraction of tropical decadal variability in the Pacific basin originates from extra-tropical stochastic forcing. These results expand previous views that highlight sources of TPDV that are internal to the tropical Pacific dynamics^22,23^ or that emerge as residuals of the ENSO cycles^17^. The framework summarized in Fig. 4 captures the growth, peak and decay of the TPDV associated with the sequence ENSO precursors (extra-tropics) → ENSO (tropics) → ENSO teleconnections (extra-tropics). These interactions between extra-tropics and tropics provide a set of testable dynamics to examine TPDV and PDV in the new generations of climate models.

## TPDV in CMIP5 Models

**Figure 5:**
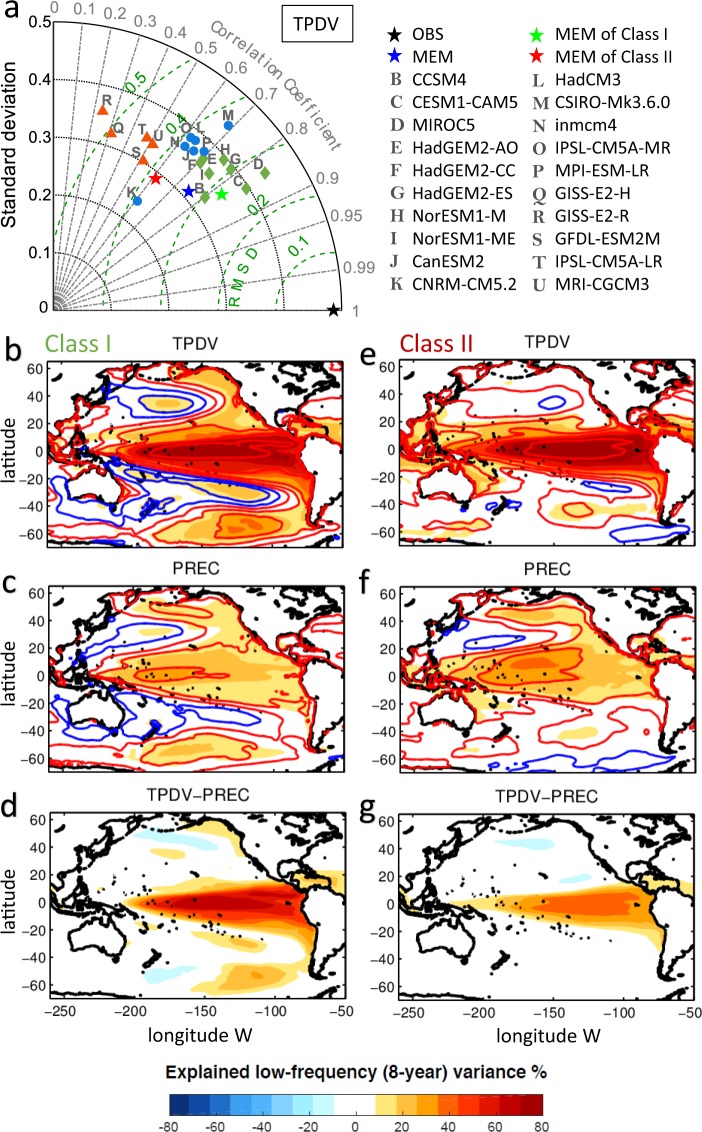
Simulation of the TPDV in CMIP5 models. (**a**) Taylor diagram for TPDV patterns simulated in CMIP5 models. The black pentagram on the x axis signifies the observational pattern as the reference point. Green dashed circles centered at the reference point represents the root mean square deviation (RMSD) and grey circles centered at the origin show the normalized standard deviation (STD). Spatial correlations are shown as cosine of the angles from the x axis. Class I (II) models are shown as green diamonds (red triangles) and other models are shown in blue points. Class I models ensemble mean (MEM) of correlation square (R^2^) between 8-year lowpass SSTa and (**b**) TPDV index, (**c**) PREC index. Class II MEM of correlation square (R2) between 8-year lowpass SSTa and (e) TPDV index, (f) PREC index. The contours in the maps show the correlation coefficients (R). Red and blue lines show the positive and negative values, respectively. Correlation of R = 0.45 (R^2^ = 20% of explained variance) or higher are significant at the 95%. (**d**) The difference map of Class I MEM TPDV variance minus Class I MEM PREC variance. (**g**) The difference map of Class II MEM TPDV variance minus Class II MEM PREC variance.

Recent observational analyses and climate model projections suggest that the variance of the precursors and ENSO may be increasing under warmer conditions, leading to stronger TPDV and PDV^45–48^. However, the ability of climate models to capture the TPDV dynamics remains a topic of debate. A computation of the TPDV and PREC spatial patterns and indices in an ensemble of climate model historical runs reveals important discrepancy from observations. Using a Taylor diagram to compare the pattern of TPDV of the climate models vs. observations reveals an important clustering in the models (Fig. 5a, Class I and Class II). The first cluster (e.g. Class I) reveals a TPDV pattern that is consistent with observations with an ENSO-like shape that extends in the extra-tropics (Fig. 5b). In contrast, the second cluster (e.g. Class II) reveals a TPDV that is mostly confined in the tropical Pacific (Fig. 5e). Further analysis of the relation between TPDV and the decadal variability pattern associated with the extra-tropical precursors (PREC, Fig. 5c,f) reveals that Class I models also capture the spatial structure of the PREC while Class II exhibit a pattern that is dominated by a North Pacific signature. However, despite the similarities of the models’ PREC pattern with observations, in both Class I and Class II, the amount of TPDV explained by the extra-tropical precursors (see different maps TPDV-PREC, Fig. 5d,g) is very small compared to the observations (Fig. 1d). This raises important questions on the dynamics that energize the TPDV and PDV in climate model. Future studies that examine and compare the mechanisms that energize the TPDV and PDV pattern (e.g. role of extra-tropical vs. tropical stochastic forcing, strength of the positive and negative feedbacks between ocean and atmosphere) are required to quantify the ability of models to reproduce realistic Pacific climate dynamics. The framework presented in this study provides an additional mechanistic hypothesis to evaluate climate models and the basis for process-based projections of the impact of a warmer climate on TPDV and PDV.

## Summary and Discussion

Often tropical Pacific low-frequency variability on timescales beyond 6–8 years, also referred in the literature as TPDV, is assumed to arise from the low-frequency residuals of ENSO and tropical dynamics^17,22,23^. However, there is a growing body of evidence suggesting that an important fraction of the TPDV is independent of the tropics and ENSO, even though ENSO is involved in the basin-scale amplification and distribution of the TPDV. Specifically, the memory associated with the seasonal progression from extra-tropics (ENSO precursors, JFM) to tropics (ENSO, OND) to extra-tropics (ENSO teleconnections, JFM + 1) allows to extract the low-frequency variability of the seasonal ENSO precursors into TPDV (see schematic and description of Fig. 4, end of Section 1). In this study, we used reanalysis data to examine the joint role of extra-tropical ENSO precursors from the North and South Pacific as independent sources of the TPDV signal, and expand on previous studies that focus on the role of ENSO precursor on ENSO and its diversity (see review by Capotondi *et al*. 2015)^49^.

Using a lag seasonal correlation analysis between ENSO in NDJ and the leading extra-tropical SLPa/SSTa in JFM, we are able to identify ENSO precursor patterns and indices for the northern and southern hemisphere that are identical to the one that emerge from more advanced methods like the use of Optimal Perturbation Patterns analysis with Linear Inverse Models (*see Supplemental Material*). This seasonal partition of the data has an important advantage from previous analyses that use monthly data, because it allows to cleanly isolate precursor indices that are independent of each other and of ENSO (Fig. 2 and Supplementary Fig. S7, Section 1). By applying an auto-regressive model of order 1 to the seasonal precursor indices we are able to extract the low-frequency variability associated with the extra-tropical precursor dynamics (e.g. the PREC index, Section 2, Fig. 1e) and estimate how much of the TPDV can be explained by PREC. We find that a significant fraction (~65%) of the tropical low-frequency variability (beyond the ENSO timescales) originates from the extra-tropical ENSO precursors (Fig. 1b,d,e, Section 1). Furthermore, we show how these precursor dynamics provide a mechanism for exchanges and synchronization of climate variance between the North and South Pacific as evident from the seasonal progression of the SSTa precursor anomalies from one hemisphere to the other (Fig. 3, Section 1) between JFM and JFM of the following year. The impacts of extra-tropical ENSO precursors on TPDV have not been quantified before using observations, nor with models which show diverse and inconsistent relations between extra-tropical ENSO precursors and TPDV (Fig. 5, Section 3). By including and quantifying together the effects of both the South and North Pacific ENSO precursors, these findings move beyond previous studies that have considered each precursor in isolation and provide a more comprehensive synthesis of the combined role of North and South Pacific extra-tropical dynamics in TPDV. The importance of short timescale process and the atmospheric noise on the tropical low-frequency variability (ENSO modulation) is also confirmed by both nonlinear coupled model and simplified linear model simulations^50^. The decadal oscillation in the extra-tropics is also shown to be reduced without tropical ENSO influence in the senility test performed by earth system model^51^.

Recent studies that examine large ensembles of individual climate models (e.g. the Community Earth System Model from the National Center for Atmospheric Research) suggest that some the ENSO precursors dynamics, specifically the Meridional Modes, may amplify under the projected climate warming scenarios for 2050–2100 and lead to a stronger TPDV^48^. This amplification can potentially lead to a synchronization of the basin-scale modes of Pacific low-frequency variability^52^ and an increase in the probability of prolonged marine heatwaves extremes in the Pacific and globally^53–55^, which are known to have dramatic consequences for marine ecosystems^56,57^. Given that climate models are necessary tools for exploring the dynamics of TPDV and its sensitivity to external forcing, future studies will have to further evaluate and understand the role of ENSO extra-tropical precursor in Pacific low-frequency climate variability.

## Methods

### Sea surface temperature, pressure and wind stress reanalysis

The observational data used in this investigation include monthly mean values of Sea Level Pressure (SLP) and 10-m wind components (U and V) from the National Centers for Environmental Prediction (NCEP) Reanalysis^58^ and Sea surface temperature (SST) from the National Oceanic and Atmospheric Administration (NOAA) Extended Reconstruction SST dataset, version3 (ERSST v3)^59^. The SLP (SST) resides on a 2.5° × 2.5° (2° × 2°) horizontal grid globally and the wind components are represented at a 1.875-degree (~1.9-degree) resolution in meridional (zonal) direction. Additional data sets are also used to further verify the results concluded from the data described above, including the Met Office Hadley Centre SLP (HadSLP2) dataset and SST (HadISST) dataset, which are on 5° × 5° and 1° × 1° horizontal grids respectively^60,61^. We also use the 40-yr European Centre for Medium-Range Weather Forecasts (ECMWF) Re-Analysis (ERA-40) 10-m wind stress dataset, which exists on a 2.5° × 2.5° horizontal grid globally. Except for the wind components, which use data from 1950 to 2000 (1958 to 2001) for NCEP reanalysis (ERA-40), we restrict the period of records to 1950–2016. In this paper, anomalies are derived by removing the mean seasonal cycle and a long-term linear trend and we focus on the Pacific basin (i.e., 100°E–60°W, 75°S–60°N).

### Model outputs

The outputs of monthly-mean SST and SLP fields from the historical simulations of 20 climate models from CMIP5 archives are used in this study^62^ (*see* Supplementary Table S1). In each model, SST and SLP outputs covering from 1861 to 2004 are interpolated onto the same grids (1° × 1° horizontal grid for the ocean and 2.5° × 2° for the atmosphere).

### Definition of El Niño Southern Oscillation (ENSO) and Decadal Variability indices

We define several indices and patterns to explore the characteristics and relationships of various climate modes during different phases of the seasonal cycle. The El Niño Southern Oscillation (ENSO) index is defined as the 1st principal component (PC) of the NOAA ERSST.v3 SST anomalies in November–December-January (NDJ) in the equatorial Pacific (5°S–5°N), which explains 85% of the variance and is associated with the season of maximum growth of ENSO anomalies. The domain for calculating ENSO index is marked as a yellow box in Fig. 2b and Supplementary Fig. S3b.

Tropical Pacific Decadal Variability (TPDV) is defined as the leading Empirical Orthogonal Functions (EOF) of 8-year low-pass SSTa in the equatorial Pacific (5°S–5°N) (explained variance is 68%), while Pacific Decadal Variability (PDV) is defined as the dominant EOF determined over the entire Pacific basin (70°S–65°N) (explained variance is 47%). The corresponding first PC time series of the tropical Pacific and the entire basin are used as the TPDV index and PDV index. The positive TPDV and PDV phases both represent the El Nino-like warm patterns. Following previous literature, we refer to these indices as “decadal variability”, however, the name of the indices is not meant to imply a preferred decadal oscillation in the data but rather the low-frequency variability on timescale of decades and longer. The existence of a preferred decadal oscillation in Pacific climate is still being debated and is not the focus of this study. Here we examine the nature of the low-frequency variance.

### Definitions of ENSO Precursors Indices and the low-frequency precursors (PREC) index

The ENSO precursor patterns, including extratropical Pacific ENSO precursor and tropical Atlantic ENSO precursor, are identified by correlating the NDJ ENSO index with SLP or SST anomalies (SLPa or SSTa) in the preceding January–February–March (JFM). The time series corresponding to SLPa and SSTa precursor patterns are obtained by projecting the spatial patterns onto the JFM anomalies. The resulting indices are defined as the northern (N) and southern (S) precursor indices *NSLP*_*pre*_, *NSST*_*pre*_, *SSLP*_*pre*_, *SSST*_*pre*_ and the Atlantic precursor index *ASST*_*pre*_. The domains for calculating the projections are marked as boxes in Fig. 2a, Supplementary Figs. S3a and S6 a. Specifically, they are 15°N–62°N, 180°E–130°W for *NSLP*_*pre*_ (blue box in Fig. 2a); 72°S–25°S, 170°E–80°W for *SSLP*_*pre*_ (red box in Fig. 2a); 10°N–55°N, 160°E–100°W for *NSST*_*pre*_ (blue box in Supplementary Fig. S3a); 60°S–15°S, 180°E–75°W for *SSST*_*pre*_ (red box in Supplementary Fig. S3a) and 25°S–25°N, 70°W–0° for *ASST*_*pre*_ (blue box in Supplementary Fig. S6a). Similarly, the successor (e.g. ENSO-induced teleconnections) patterns and indices are obtained by correlating the NDJ ENSO index with the SLPa or SSTa of JFM in the following year (JFM + 1), and are defined as *NSLP*_*post*_, *NSST*_*post*_, *SSLP*_*post*_ and *SSST*_*post*_. The domains for calculating the successor indices are also marked as boxes in Fig. 2c and Supplementary Fig. S3c. Specifically, they are 15°N–64°N, 120°E–100°W for *NSLP*_*post*_(blue box in Fig. 2c); 75°S–15°S, 135°E–70°W for *SSLP*_*post*_(red box in Fig. 2c); 10°N–60°N, 120°E–100°W for *NSST*_*post*_ (blue box in Supplementary Fig. S3c) and 60°S–15°S, 180°E–75°W for *SSST*_*post*_ (red box in Supplementary Fig. S3c). The precursor patterns and indices obtained by this simple seasonal lead-lag correlation approach are also recovered using more rigorous optimal perturbation calculations with MLR method and a linear inverse modeling (*see Supplemental Material*) - an approach that is discussed extensively in the literatures^63,64^.

A linear model that weighs precursor indices separately is used to reconstruct ENSO: $ENS{O}_{re}=a\times NSL{P}_{pre}$ $+b\times SSL{P}_{pre}$ or $ENS{O}_{re}=a\times NSS{T}_{pre}+b\times SSS{T}_{pre}$, where *a* +* b* = 1, and the choice of *a*, *b*. maximizes the correlation between *ENSO*_*re*_ and ENSO. Here the correlation coefficients between these two *ENSO*_*re*_ indices and ENSO are highest when a = 0.5 and b = 0.5. The low-frequency PREC index associated with the reddening of the ENSO precursors is obtained by applying an auto-regressive model of order 1 (AR-1) forced with the ENSO precursor timeseries: $ENS{O}_{Precursors}=0.5\times NSS{T}_{pre}+0.5\times SSS{T}_{pre}$,

1 $$\frac{dPREC(t)}{dt}=ENS{O}_{Precursors}(t)-\frac{PREC(t)}{\tau }.$$

The integration of the ENSO precursors forcing term provides a formal quantification of the reddening (e.g. filter and phase shift of the low-frequency variability) following the Hasselmann (1976) model^44^. In this formulation, the reddening depends only on the memory of the coupled system, which is captured by the parameter *τ*. Physically, this memory represents the decay timescale associated with the process ENSO-Precursors (JFM) → ENSO (OND) → ENSO Successors (JFM+1) → extra-tropical decay of SSTa, which is about 1–1.5 years. When comparing the PREC and TPDV indices we find that the correlation between indices is not very sensitive to *τ* (R = 0.78–0.82) and has a maximum at *τ* = 1.2 *years*. The timestep *dt* used in the AR-1 model is one season. Consistent with the AR-1 model assumption that the forcing term is “white noise” (e.g. no auto-correlation), the auto-correlation function of the *ENSO*_*Precursors*_ shows an insignificant lag-1 auto-correlation (Fig. S7).

### Statistical significance testing

We applied significance tests to the analyses in this paper. The significance of correlations and regressions are estimated based on the Probability Density Function (PDF) calculated using the Monte Carlo method. For each test, the PDF is inferred from 100,000 pairs of random red noise samples with the same lag 1 correlation as the data.

## Supplementary information

Supplementary information

## Data Availability

All data analyzed during this study are included in this article (and its Supplementary Information file).
