# Supplementary material for: The impacts of Extra-tropical ENSO Precursors on Tropical Pacific Decadal-scale Variability
Source: Sci Rep. 2020 Feb 20;10:3031. doi: 10.1038/s41598-020-59253-3 (PMC7033232; doi:10.1038/s41598-020-59253-3)
Supplement: Supplementary file 1 — Supplementary information [file 41598_2020_59253_MOESM1_ESM.pdf]

The impacts of Extra-tropical ENSO Precursors on Tropical Pacific Decadal-scale Variability

*Yingying Zhao and Emanuele Di Lorenzo**

Program in Ocean Science and Engineering, Georgia Institute of Technology, Atlanta, USA

Supplemental Material

*Corresponding Author

Emanuele Di Lorenzo

Program in Ocean Science and Engineering

Georgia Institute of Technology

311 Ferst Drive NE, Atlanta, GA 30332, USA

edl@gatech.edu

+1 (404) 788-8035

Supplemental Material

Optimal perturbation patterns of ENSO in the extra-tropics

Previous studies have successfully used Linear Inverse Model (LIM) to approximate the tropical ocean-atmospheric system[1,2,3]. To further confirm the ENSO precursor dynamics, we develop a LIM to extract from observations the optimal perturbation patterns in the extra-tropics that energize ENSO. The dynamical system is represented following ref.[3] as

$$\frac{\mathrm{d}\mathbf{x}}{\mathrm{dt}} = \boldsymbol{L}\mathbf{x} + \xi.$$

Here the evolution of the system is controlled by the linear dynamical evolution operator $\boldsymbol{L}$ and the noise forcing ξ. $\boldsymbol{L}$ can be determined based on the covariance of the state vector $\mathbf{x}$ as described in ref.[4,2]:

$$\boldsymbol{L} = {\tau_0}^{-1} ln\{\boldsymbol{C}(\tau_0)\boldsymbol{C}(0)^{-1}\}$$

where $\boldsymbol{C}(\tau_0)$ and $\boldsymbol{C}(0)$ are the covariance matrices at lag τ_0 and 0. In this work, we chose $\tau_0 = 3$.

The state vector $\mathbf{x}$ is constructed as

$$\mathbf{x} = \begin{bmatrix} \boldsymbol{T_T} \\ \boldsymbol{T_N} \\ \boldsymbol{T_S} \end{bmatrix},$$

Where $\boldsymbol{T_T}$, $\boldsymbol{T_N}$ and $\boldsymbol{T_S}$ are the leading 10 PCs of SST anomalies of tropical Pacific ($5°\mathrm{S} - 5°\mathrm{N}$), North Pacific ($5°\mathrm{N} - 60°\mathrm{N}$) and South Pacific ($60°\mathrm{S} - 5°\mathrm{S}$) correspondingly. The SST anomalies were calculated using the monthly NOAA SST from 1950 to 2016 by removing the long-term monthly mean. We applied 3-month running averaging and removed the linear trend from the data. Anomalies from each region were normalized by the domain-averaged climatological root-mean-square amplitude.

Ref.[2, 3] showed that the LIM can be used to identify the "optimal" initial condition for maximizing the

amplification of the tropical SST anomalies. Using the same approach, we obtained the optimal initial condition via the singular vector decomposition (SVD) of the system propagator $\boldsymbol{G}(\tau) = exp(\boldsymbol{L}\tau)$ to maximize L2 norm of tropical SST anomalies (i.e., $\boldsymbol{T_T}^{\boldsymbol{T}}\boldsymbol{T_T}$). Here we chose $\tau = 8$ month, by which the growth of the initial anomalies are maximized in LIM. This is also consistent with the lag between precursor and the ENSO time series in the observation (9-10 months). The optimal initial conditions in the North and South Pacific based on our LIM are showed in Figure S1 a and b. The typical foot printing pattern in the North Pacific and the quadrupole structure close to South America share common features with the precursor pattern inferred from the correlation analysis between SST anomalies and ENSO time series (Figure S3a). The projection of SST anomalies onto North Pacific and South Pacific optimal initial conditions in the corresponding regions to compute $NSST_{pre}$ and $SSST_{pre}$ (blue box and red box in Figure S3a) are called $NSST_{LIM}$ and $SSST_{LIM}$ indices, which show high correlation with the precursor indices $NSST_{pre}$ and $SSST_{pre}$ (R = 0.86 for the North Pacific and R = 0.72 for the South Pacific) (Figure S1 c and d).

ENSO precursors based on multivariable linear regression

Another approach to infer the precursor dynamics is through multivariable linear regression (MLR). Assuming that the tropical variabilities is linearly correlated with the preceding extra-tropical anomalies, the linear regression equation can be written as

$$\mathrm{y} = \mathrm{Ex} + \mathrm{r},$$

where y is the leading 10 PCs of SST anomalies of tropical Pacific (5°S – 5°N) in NDJ, x is the leading 10 PCs of SST / SLP anomalies of North (5°N – 60°N) / South (60°S – 5°S) Pacific in JFM of the same year, E is the matrix of regression coefficients and r is the residual. With the least square method, E can be estimated using the PC time series. The SVD of E yields a dominant pair u_1, v_1 and the largest singular value λ_1 with

$$\mathrm{E}v_1 = \lambda_1 u_1.$$

v_1 represents the precursor pattern that correlated with the maximum tropical anomalies with the spatial feature indicated by u_1, which coincides with ENSO in this practice (not show here). The spatial patterns and time series calculated using this MLR method are shown in Figure S2. The patterns based on MLR share similar pattern with the correlation map between SLPa / SSTa in JFM and the NDJ ENSO index (Figure 2a and Figure S3a) in corresponding regions. The MLR precursor indices ($NSST_{MLR}, SSST_{MLR}, NSLP_{MLR}, SSLP_{MLR}$) can be calculated by projecting the SSTa of JFM onto the precursor patterns in certain regions (boxes shown in Figure 2a and Figure S3a). These MLR precursor indices show high correlation with the precursor indices inferred from the correlation analysis (Figure S2 b, c, e and f).

Reference

1. Penland, C. & Matrosova, L. A Balance Condition for Stochastic Numerical Models with Application to the El Niño-Southern Oscillation. *J. Clim.* **7(7)**, 1352-1372 (1994).
2. Penland, C. & Sardeshmukh, P. D. The optimal-growth of tropical sea-surface temperature anomalies. *J. Clim.* **8(8)**, 1999–2024 (1995).
3. Newman, M., Alexander, M. A. & Scott, J. D. An empirical model of tropical ocean dynamics. *Clim. Dyn.* **37.9-10(2011)**, 1823-1841 (2011).
4. Penland C. Random Forcing and Forecasting Using Principal Oscillation Pattern Analysis. *Mon. Wea. Rev.* **117**, 2165 (1989).

Table

Institute and country	Model name	Class
Canadian Centre for Climate Modeling and Analysis (CCCma), Canada	CanESM2	
National Center for Atmospheric Research (NCAR), United States	CCSM4	I
National Science Foundation, Department of Energy, National Center for Atmospheric Research (NSF-DOE-NCAR),United States	CESM1-CAM5	I
Centre National de Recherches Meteorologiques / Centre Europeen de Recherche et Formation Avanceesen Calcul Scientifiqu (CNRM-CERFACS), France	CNRM-CM5.2	
CSIRO in collaboration with the Queensland Climate Change Centre of Excellence (CSIRO-QCCCE), Australia	CSIRO-Mk3.6.0	
NOAA Geophysical Fluid Dynamics Laboratory (GFDL), United States	GFDL-ESM2M	II
NASA Goddard Institute for Space Studies (GISS), United States	GISS-E2-R<br>GISS-E2-H	II<br>II
Met Office Hadley Centre (additional HadGEM2-ES realizations contributed by Instituto Nacional de Pesquisas Espaciais), United Kingdom	HadCM3<br>HadGEM2-AO<br>HadGEM2-CC<br>HadGEM2-ES	 I<br>I<br>I
Institute for Numerical Mathematics (INM), Russia	INMCM4	
Institute Pierre-Simon Laplace (IPSL), France	IPSL-CM5A-LR<br>IPSL-CM5A-MR	II
Atmosphere and Ocean Research Institute (AORI; The University of Tokyo), National Institute for Environmental Studies (NIES), and Japan Agency for Marine-Earth Science and Technology (JAMESTEC), Japan	MIROC5	I
Max Planck Institute for Meteorology (MPI-M), Germany	MPI-ESM-LR	
Meteorological Research Institute (MRI), Japan	MRI-CGCM3	II
Norwegian Climate Centre (NCC), Norway	NorESM1-M<br>NorESM1-ME	I<br>I

Table S1 List of models analyzed in the present study from the CMIP5 database. Model's institute and country, model name and Class of the model are given.

Figures

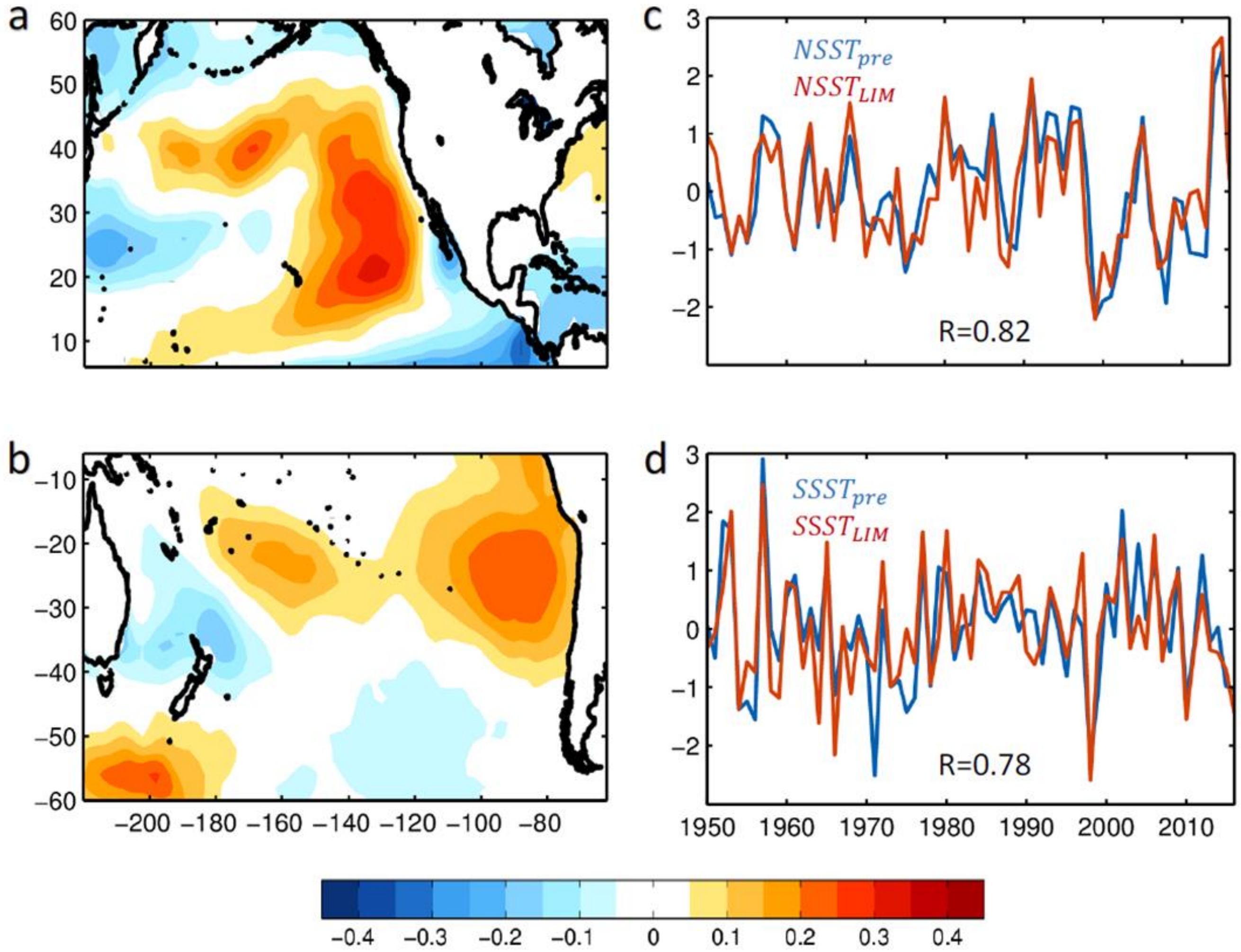

Figure S1: ENSO optimal perturbation patterns in the extra-tropics obtained from the Linear Inverse Model (LIM) (see Supplemental Material). Patterns show the SSTa ENSO optimal initial condition in (a) North Pacific and (b) South Pacific. Comparison of (c) $NSST_{LIM}$ index (red line) and $NSST_{pre}$ index (blue line), (d) $SSST_{LIM}$ index (red line) and $SSST_{pre}$ index (blue line). Also indicated are the correlation coefficients between indices, which pass the 99% confidence level.

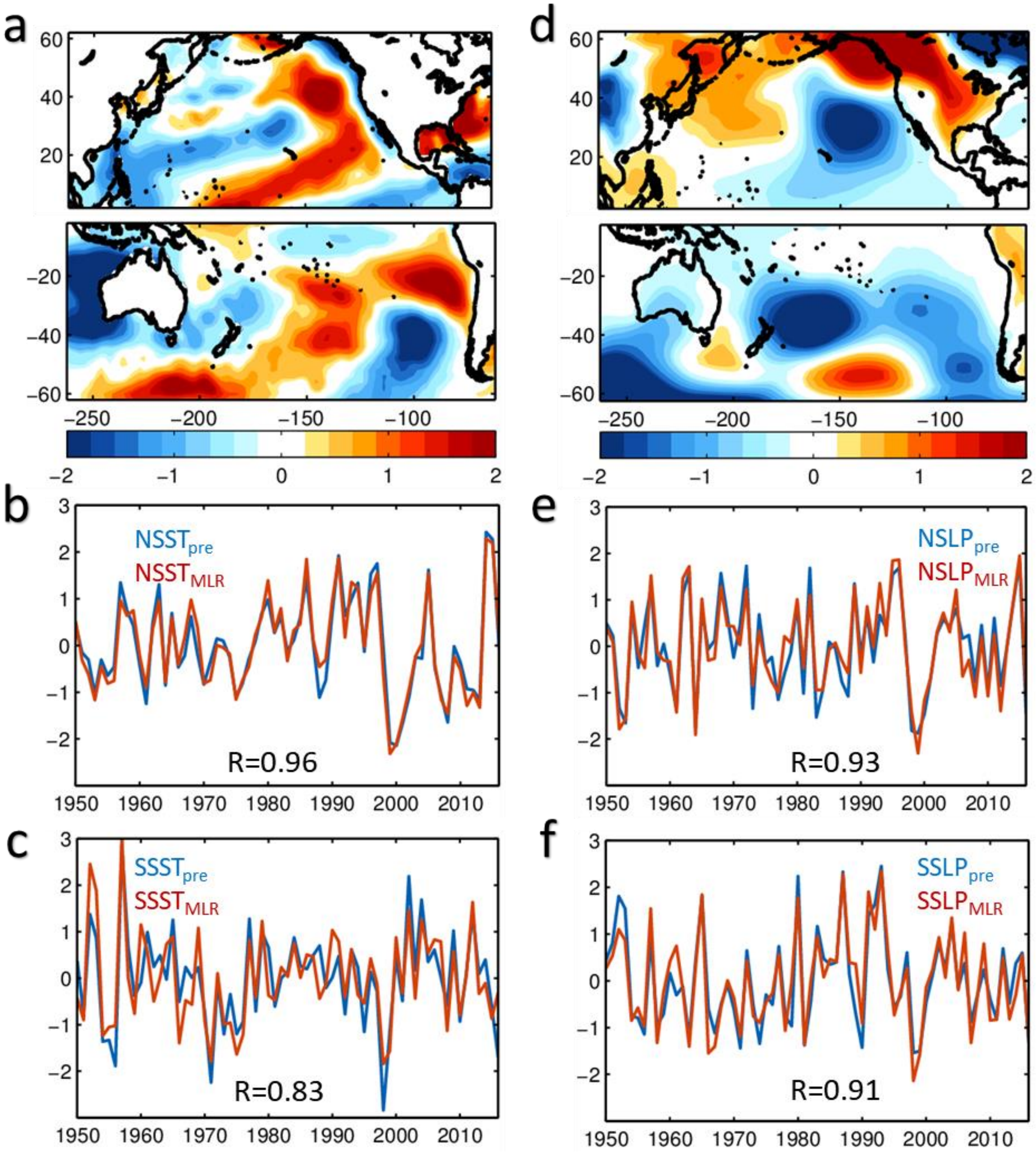

Figure S2: Extra-tropical precursor patterns that correlated with the maximum tropical SST anomalies obtained from the multivariable linear regression (MLR) and the corresponding precursor indices (see Supplemental Material). Patterns show the extra-tropical precursor patterns in (a) SSTa field and (d) SLPa field. Comparison of (b) $NSST_{MLR}$ index (red line) and $NSST_{pre}$ index (blue line), (c) $SSST_{MLR}$ index (red line) and $SSST_{pre}$ index (blue line), (e) $NSLP_{MLR}$ index (red line) and $NSLP_{pre}$ index (blue line), (f) $SSLP_{MLR}$ index (red line) and $SSLP_{pre}$ index (blue line). Also indicated are the correlation coefficients between indices, which pass the 99% confidence level.

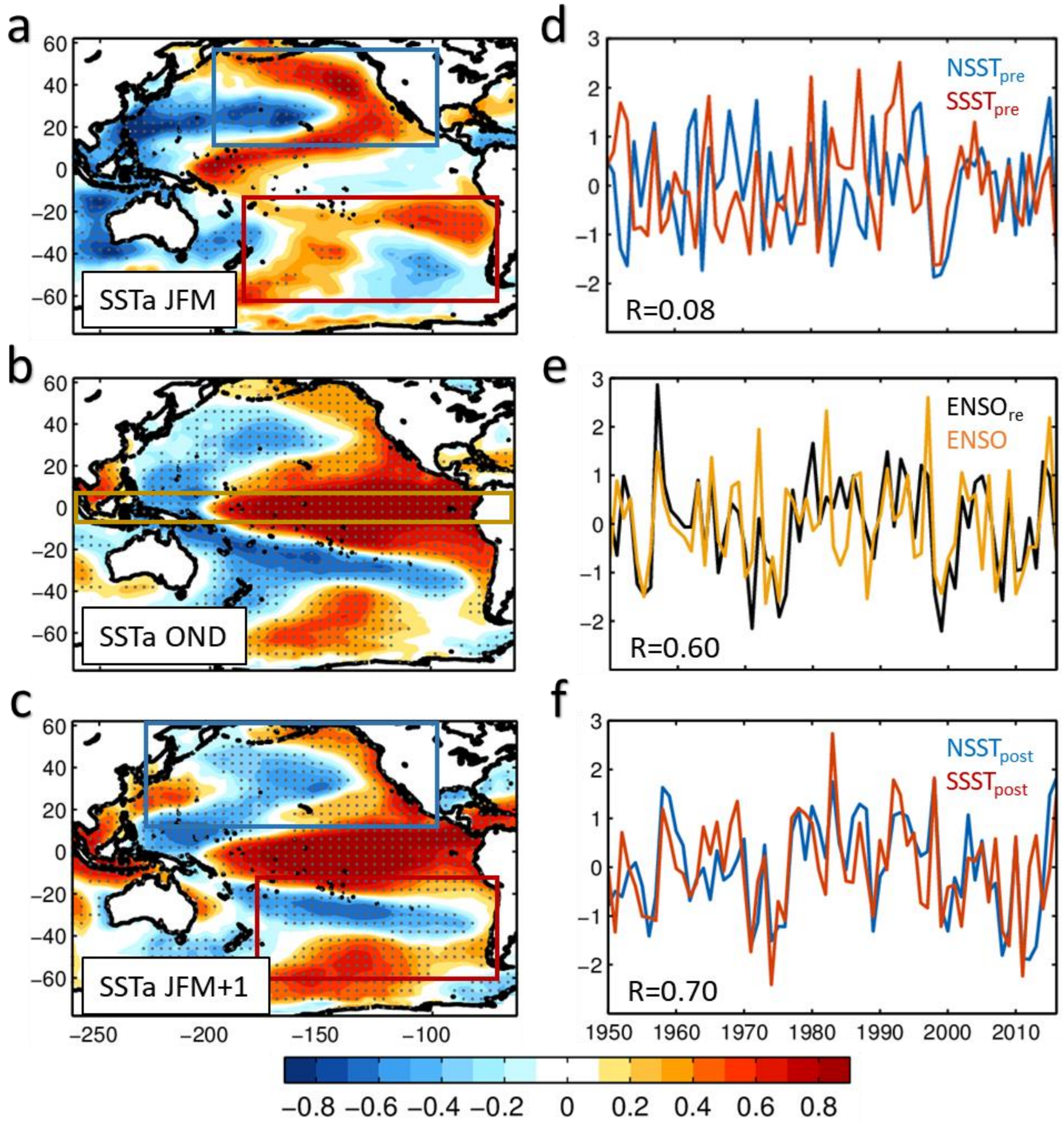

Figure S3: Oceanic ENSO precursor, ENSO and ENSO successor patterns and the corresponding indices. Patterns are obtained by correlating NDJ ENSO index with (a) NOAA SSTa in JFM, (b) NOAA SSTa in OND, and (c) NOAA SSTa in the following JFM. Note that the correlations in figure (a) are multiplied by 2. Grey dots show the regions that pass the 90% confidence level. The boxes in figure (a), (b) and (c) show the domains for calculating the corresponding indices in figure (d), (e) and (f), respectively. More details are described in Method. Comparison of (d) $NSST_{pre}$ index (blue line) and $SSST_{pre}$ index (red line), (e) ENSO index (yellow line) and ENSO$_{re}$ index (black line): ENSO$_{re} = 0.5 \times NSST_{pre} + 0.5 \times SSST_{pre}$, (f) $NSST_{post}$ index (blue line) and $SSST_{post}$ index (red line). Also indicated are the correlation coefficients between indices. Correlation coefficients in figure (e) and (f) pass the 99% confidence level.

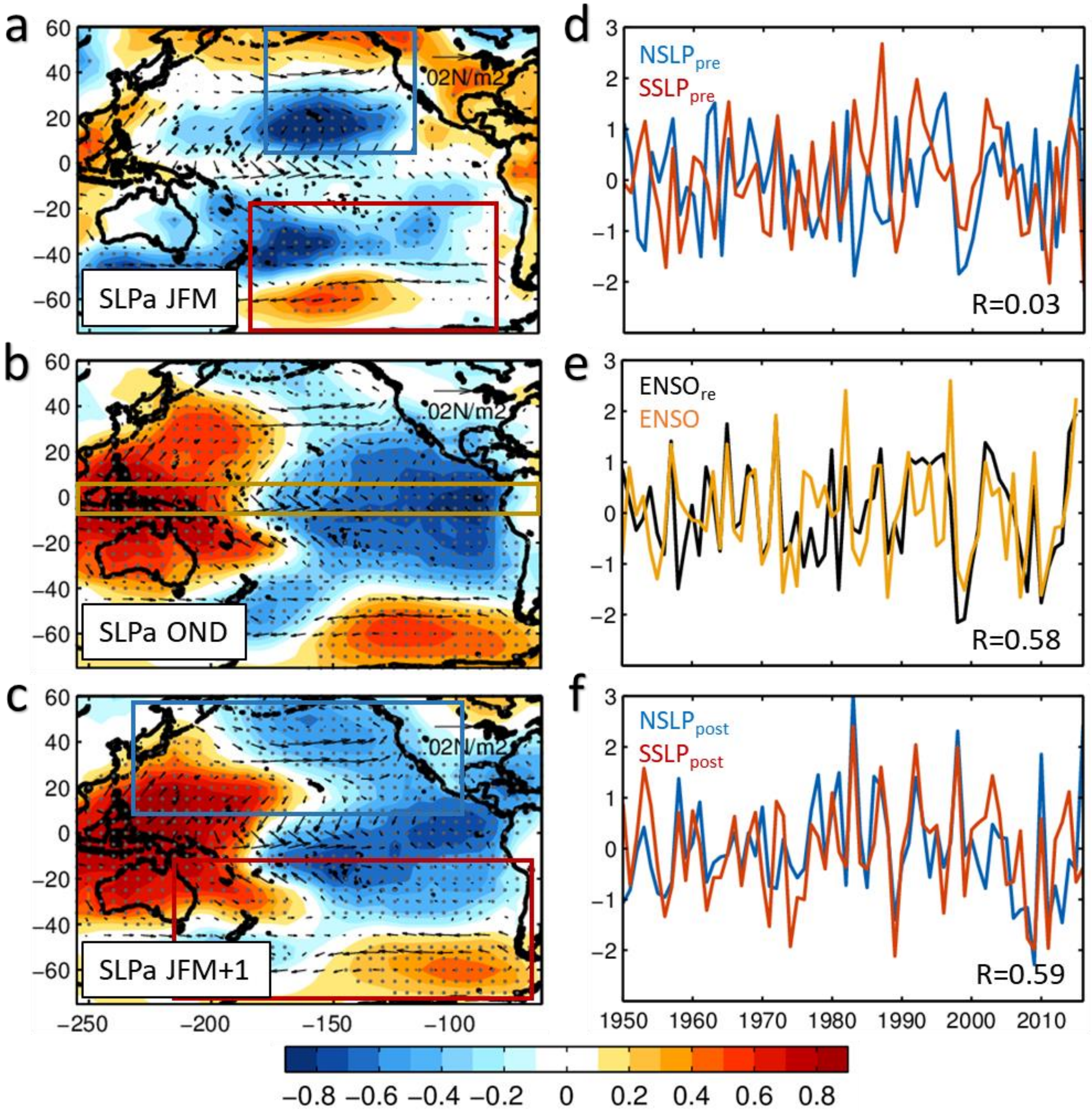

Figure S4: Atmospheric ENSO precursor, ENSO and ENSO successor patterns and the corresponding indices. Similar to Figure 2, but the observational data used here are Hadley SLP and ERA40 wind stress. Correlation coefficients in figure (e) and (f) pass the 99% confidence level.

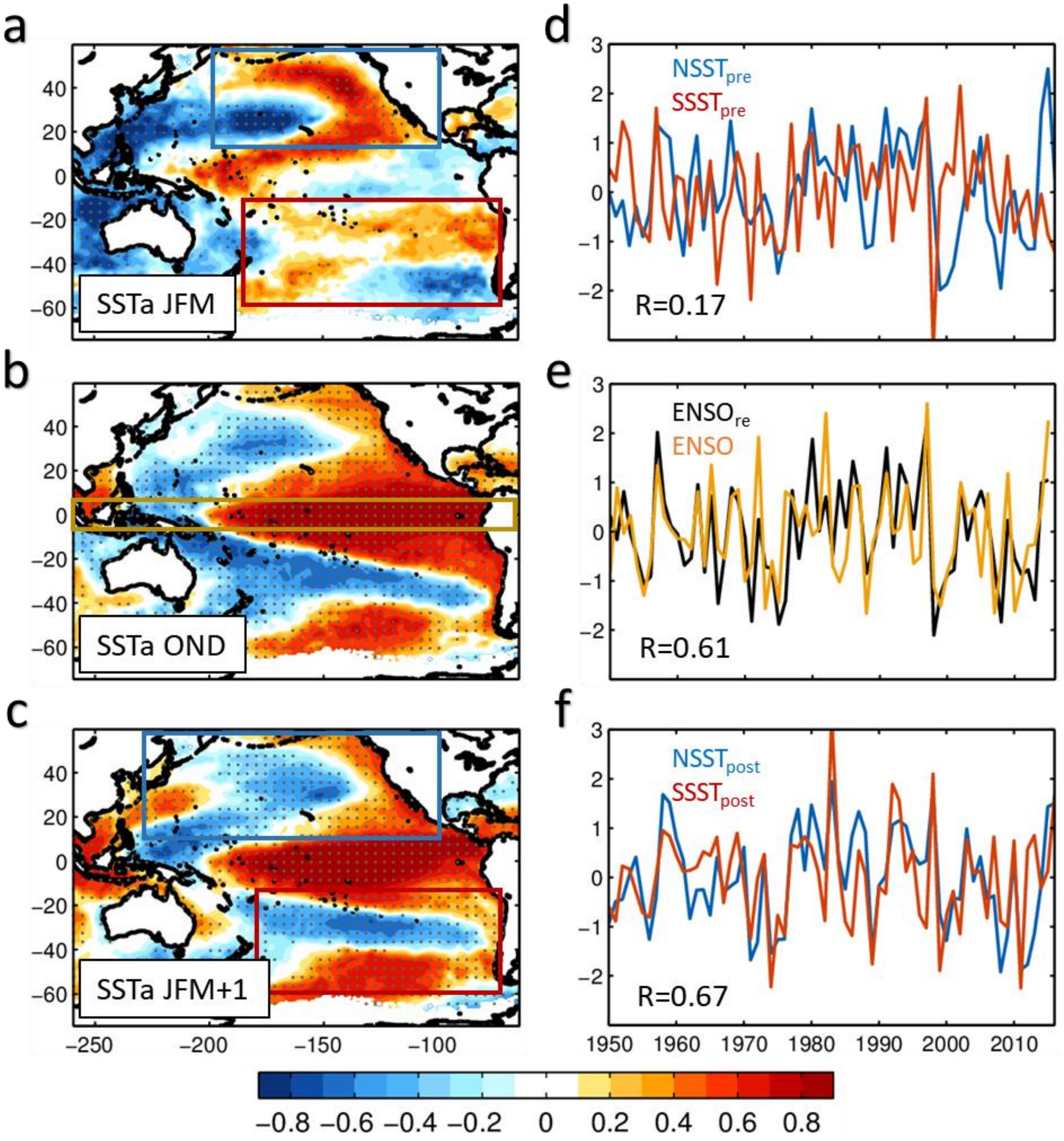

Figure S5: Oceanic ENSO precursor, ENSO and ENSO successor patterns and the corresponding indices. Similar to Figure S3, but the observational data used here are Hadley SST and ERA40 wind stress. Correlation coefficients in figure (e) and (f) pass the 99% confidence level.

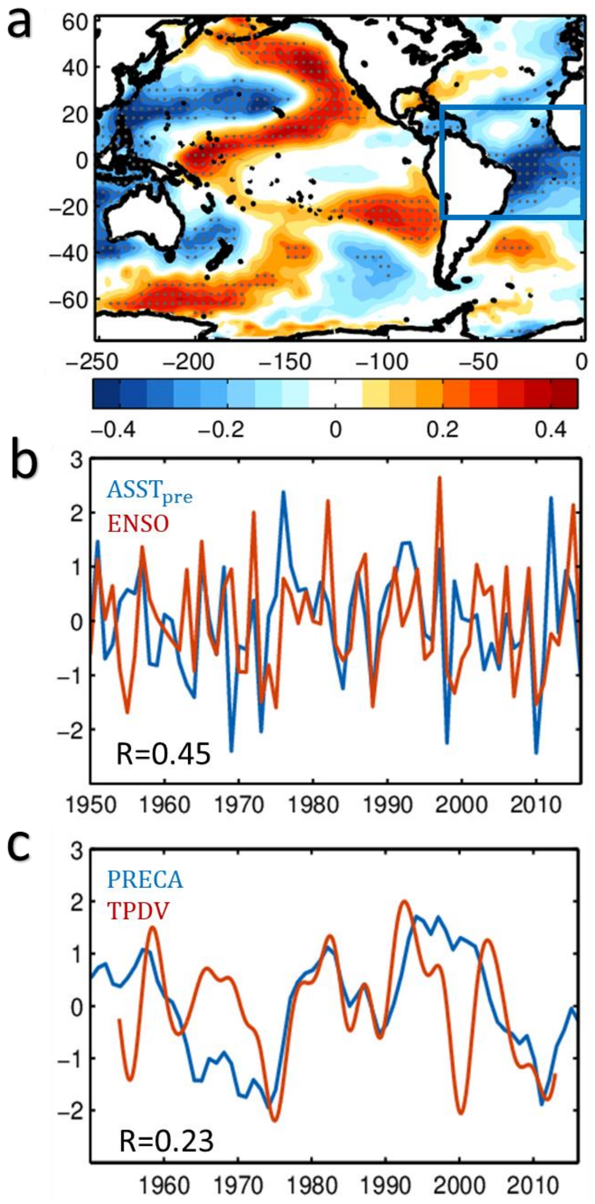

Figure S6: Atlantic ENSO precursor pattern and the corresponding indices. (a) Correlation map between ENSO index and NOAA SSTa in JFM. Grey dots show the regions that pass the 90% confidence level. The blue boxes in figure (a) show the domains for calculating the $ASST_{pre}$ index . More details are described in Method. Comparison of (b) $ASST_{pre}$ index (blue line) and $ENSO$ index (red line), (c) PRECA index (blue line) and $TPDV$ index (red line). The low-frequency PRECA index is obtained by applying an auto-regressive model of order 1 (AR-1) forced with the Atlantic precursor timeseries. $\frac{dPRECA(t)}{dt} = ASST_{pre} - \frac{PRECA(t)}{\tau}$, which τ=1.2. Also indicated are the correlation coefficients between indices. Correlation coefficient in figure (b) passes the 99% confidence level.

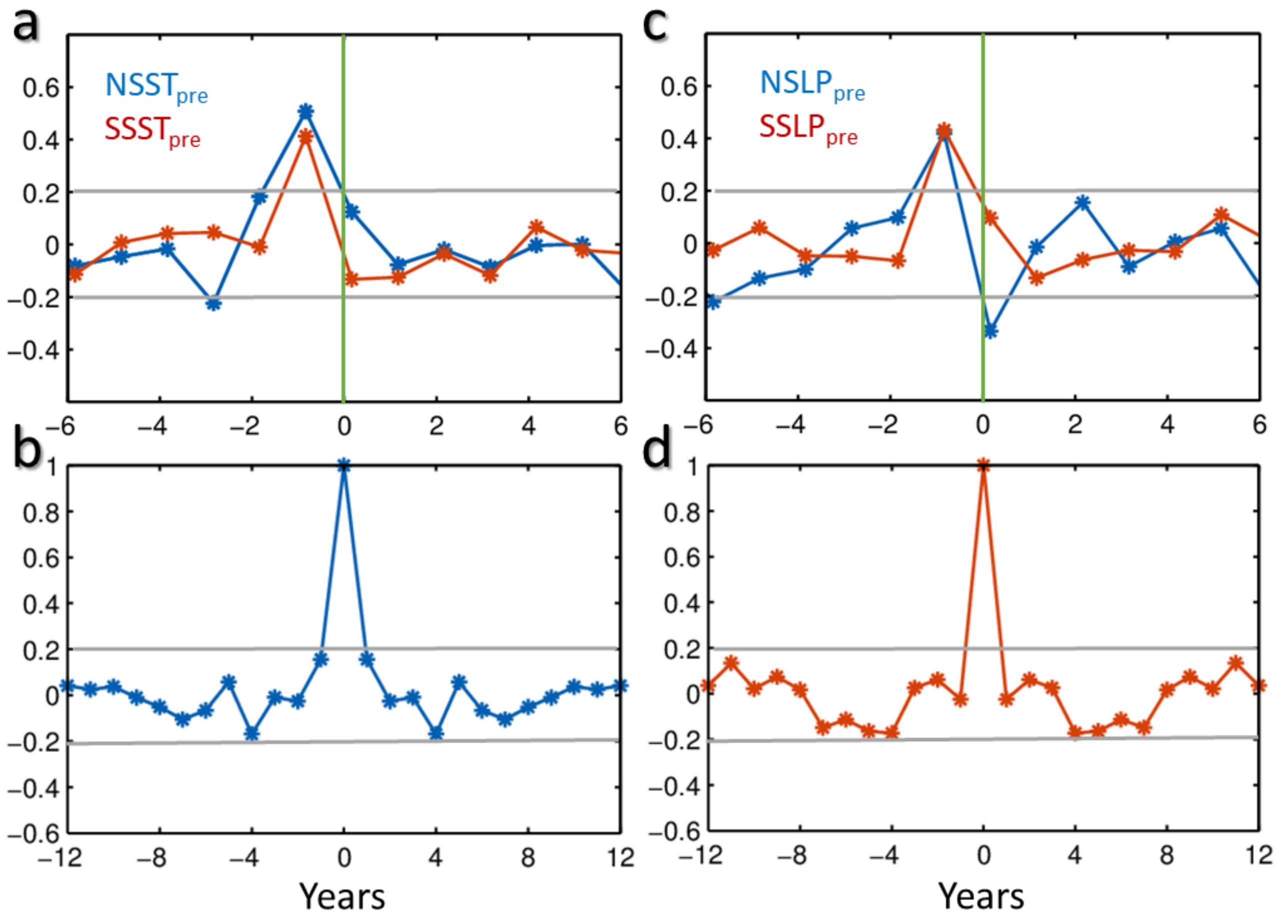

Figure S7: Cross correlations between NDJ ENSO index and precursor indices and auto-correlations of $ENSO_{precursors}$ indices. Cross correlations between NDJ ENSO index and (a) $NSST_{pre}$ index (blue line) and $SSST_{pre}$ index (red line); (c) $NSLP_{pre}$ index (blue line) and $SSLP_{pre}$ index (red line). Correlations in the left quadrant of each panel imply that precursors indices lead ENSO, in the right quadrant ENSO leads. Auto-correlations of $ENSO_{precursors}$ indices: (b) $ENSO_{Precursors} = 0.5 \times NSST_{pre} + 0.5 \times SSST_{pre}$, (d) $ENSO_{Precursors} = 0.5 \times NSLP_{pre} + 0.5 \times SSLP_{pre}$.

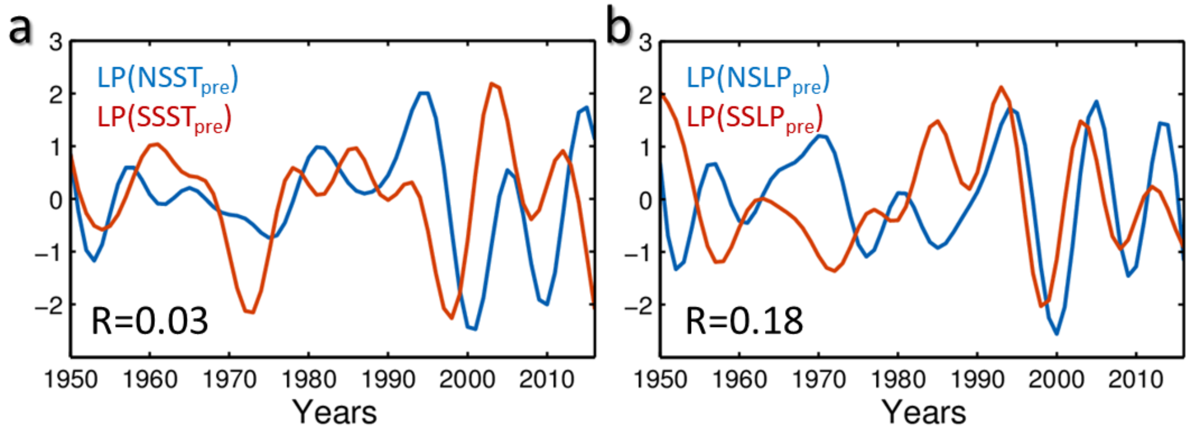

Figure S8: 8-year low pass precursor indices. (a) 8-year low pass $NSST_{pre}$ index (blue line) and 8-year low pass $SSST_{pre}$ index (red line); (b) 8-year low pass $NSLP_{pre}$ index (blue line) and 8-year low pass $SSLP_{pre}$ index (red line). Also indicated are the correlation coefficients between the indices.
